# Editorial Comment from Dr Fukuhara and Dr Nonomura to A case of hypogonadtropic hypogonadism due to hypophysitisdiscovered by secondary male infertility

**DOI:** 10.1002/iju5.12556

**Published:** 2022-11-04

**Authors:** Shinichiro Fukuhara, Norio Nonomura

**Affiliations:** ^1^ Department of Urology Osaka University Graduate School of Medicine Suita Japan

The authors reported a case of male hypogonadtropic hypogonadism (MHH) due to hypophysitis discovered by secondary male infertility.^1^ Adult‐onset MHH is a treatable condition in the practice of male infertility, and it is important to properly diagnose it. Reproductive health care providers and urologists should be familiar with MHH, as in this case, the patient may present with a decreased libido and male infertility. As noted by the authors, adult‐onset MHH is often the outcome of posttreatment pituitary disease or trauma. We report a case of MHH caused by lymphocytic hypophysitis, which is itself a rare disorder, and it is important to note that gonadotropin deficiency can be caused by pituitary inflammation as well as other pituitary diseases that can lead to MHH. Other pituitary disorders may also affect only the gonadotropin‐producing cells without affecting the production of adrenocorticotropic hormone released from the anterior pituitary gland.

In the case of congenital (pre‐pubertal‐onset) MHH, testicular volume is often less than 4 mL and/or shows a cryptorchid testis. This case was diagnosed as an acquired MHH because the testicular volume was 10 mL. It has been reported that the larger the testicular volume at diagnosis, the better the response to hormone therapy.^2^ Gonadotropin replacement therapy is the treatment of MHH. Luteinizing hormone (LH) stimulates testosterone production in the testes, but LH itself is not suitable for clinical use due to its very short half‐life. Human chorionic gonadotropin (hCG) exhibits similar kinetics to LH in vivo and is pharmacologically suitable as a replacement therapy. If both hCG alone does not stimulate spermatogenesis, the addition of a follicle‐stimulating hormone (FSH) preparation may be considered. Alternatively, in cases where infertility is the primary complaint, the combination of hCG and FSH has been reported to have better results from the outset.^3^

Adult‐onset MHH often responds well to treatment.

In this case, 17 months of HCG replacement therapy seems to have improved libido, probably due to the normalization of testosterone levels, but sperm have not yet appeared in the semen.

We hope that the addition of rFSH supplementation will help sperm to appear. Like MHH caused by other pituitary diseases, MHH caused by lymphocytic hypophysitis appears to be irreversible, but whether MHH caused by lymphocytic hypophysitis is reversible or not also awaits future reports.

## Author contributions

Shinichiro Fukuhara: Conceptualization; writing – original draft. Norio Nonomura: Conceptualization; writing – review and editing.

## Conflict of interest

The authors declare no conflict of interest .
